# Erste Erfahrungen bei der Umsetzung der EU-Verordnung 536/2014 (CTR) aus Sicht der nichtkommerziellen akademischen Forschung

**DOI:** 10.1007/s00103-022-03632-w

**Published:** 2022-12-16

**Authors:** Christine Fuhrmann, Corinna Reineke, Britta Lang, Xina Grählert

**Affiliations:** 1 Studienzentrale SZB, Institut für Klinische Chemie und Klinische Pharmakologie, Rheinische Friedrich-Wilhelms-Universität Bonn, Venusberg-Campus 1, 53127 Bonn, Deutschland; 2 Zentrum Klinische Studien, Universitätsklinikum Freiburg, Medizinische Fakultät, Albert-Ludwigs-Universität Freiburg, Freiburg, Deutschland; 3 Medizinische Fakultät Carl Gustav Carus, Koordinierungszentrum für Klinische Studien Dresden, Technische Universität Dresden, Dresden, Deutschland

**Keywords:** Clinical Trials Regulation (CTR), Clinical Trials Information System (CTIS), Investigator-initiated Trial (IIT), Akademische Forschungseinrichtungen, Erfahrungsbericht, Clinical Trials Regulation (CTR), Clinical Trials Information System (CTIS), Investigator-initiated trial (IIT), Academia, Experience report

## Abstract

Mit der Implementierung der neuen EU-Verordnung 536/2014 (Clinical Trials Regulation – CTR) zum 31.01.2022 sollen Genehmigung und Durchführung klinischer Prüfungen mit Humanarzneimitteln innerhalb der Europäischen Union (EU) harmonisiert werden. Die Genehmigung erfolgt über das elektronische Portal CTIS (Clinical Trials Information System) der Europäischen Arzneimittel-Agentur (EMA). Neben kommerziellen Sponsoren sind im Rahmen von wissenschaftsinitiierten klinischen Prüfungen (IIT) auch Sponsoren an akademischen Einrichtungen von der Implementierung der CTR betroffen. Zahlreiche Änderungen in der Prozesslandkarte für regulierte Arzneimittelstudien sind notwendig.

Neue Aspekte betreffen die generelle Nutzerstruktur und das Rechtekonzept des CTIS. Anforderungen, die bislang nur für Prüfpräparate/Placebo galten, gelten nun auch für Hilfspräparate. In der EU noch nicht zugelassene Prüf- und Hilfspräparate müssen in der Arzneimitteldatenbank XEVMPD registriert werden. Weitere wesentliche Neuerungen sind die Meldung von „schwerwiegenden Verstößen“, die Veröffentlichung relevanter Studiendokumente, die Einführung einer „laienverständlichen Zusammenfassung“, die Archivierungsdauer von 25 Jahren, das Konzept der „minimalinterventionellen klinischen Prüfung“ und die Möglichkeit einer Co-Sponsorenschaft.

Die ersten Erfahrungen bei der Antragstellung zeigen, dass das neue System noch weiter verbessert werden muss. Dies betrifft z. B. die EU-weite Harmonisierung der Anforderungen und die Behebung technischer Mängel. Mittel- und langfristig dürften aber Erleichterungen in Bezug auf regulatorische Prozesse spürbar sein. Hier sind intensivierte Absprachen mit nationalen Oberbehörden und Ethikkommissionen, effektives Wissensmanagement und verbesserte Kommunikation gefragt.

## Abstract (en)

With the implementation of the new EU Regulation 536/2014 (Clinical Trials Regulation – CTR) on 31 January 2022, the approval and conduct of clinical trials with medicinal products for human use are to be harmonized within the European Union (EU). Approval is granted via the electronic Clinical Trials Information System (CTIS) portal of the European Medicines Agency (EMA). In addition to commercial sponsors, sponsors at academic institutions are also affected by the implementation of the CTR in the context of investigator-initiated clinical trials (IITs). Numerous changes in the process map for regulated drug trials are necessary.

New aspects concern the general user structure and the role and permission concept of CTIS. Requirements that previously applied only to investigational medicinal products/placebo now also apply to auxiliary medicinal products. Investigational and auxiliary medicinal products not yet approved in the EU must be registered in the XEVMPD drug database. Other significant changes include the reporting of “serious breaches,” the publication of relevant study documents, the introduction of a “summary in layman’s terms,” the archiving period of 25 years, the implementation of “low intervention clinical trials,” and the possibility of co-sponsorship.

First experience with the application process shows that the new system needs to be further improved. This concerns, for example, the EU-wide harmonization of requirements and the elimination of technical deficiencies. In the medium and long term, however, simplifications with regard to regulatory processes should be noticeable. What is needed here are intensified agreements with national higher authorities and ethics committees, effective knowledge management, and improved communication.

## Einleitung

Ziel der EU-Verordnung 536/2014 (Clinical Trials Regulation – CTR; [1]), die zum 31.01.2022 anwendbar wurde, ist es, die Verfahren für die Antragstellung und Durchführung klinischer Prüfungen mit Humanarzneimitteln innerhalb der Europäischen Union (EU) zu harmonisieren. Anstelle einer Vielfalt nationaler Prozesse erfolgt nunmehr eine zentrale Einreichung bei Behörden und Ethikkommissionen über das elektronische Portal „Clinical Trials Information System“ (CTIS) der Europäischen Arzneimittel-Agentur (EMA; [2]) nach einheitlichen Vorgaben. Eine Genehmigung wird im konsolidierten Verfahren über alle betroffenen Mitgliedstaaten erteilt. Für alle Sponsoren, Industrie wie auch akademische Forschungseinrichtungen, gelten dieselben Regelungen. Strenge Fristen sollen für eine zügige Bearbeitung der Anträge durch alle Beteiligten sorgen. Soweit die Vision.

In der Praxis stellt die Implementierung des neuen Antragsverfahrens die Beteiligten jedoch vor große Herausforderungen: So müssen nach der Durchführung von Gap-Analysen Prozesse vollständig überarbeitet und notwendige Ressourcen zugewiesen werden. Daneben müssen laufende klinische Prüfungen, falls sie nicht innerhalb einer Frist von 3 Jahren (bis 31.01.2025) beendet sein werden,^1^ in das EU-Portal CTIS überführt werden.

Da die CTR unmittelbar geltendes Recht in allen Mitgliedstaaten ist, finden seit Februar 2022 einzelne nationale Gesetze und Verordnungen, die nicht auf Öffnungsklauseln beruhen, keine Anwendung mehr. In Deutschland betrifft dies insbesondere die GCP-Verordnung^2^ [3]. Diese gilt nur noch innerhalb der Übergangsfristen für nach bisherigem Recht eingereichte Anträge und laufende klinische Prüfungen. Für akademische Sponsoren und Antragsteller stellt all dies aufgrund der knappen personellen Ressourcen eine große Herausforderung dar. Im Folgenden sollen aus Sicht der akademischen Forschungseinrichtungen Aspekte der Umsetzung der CTR aufgezeigt und erste Erfahrungen mit CTIS und dem dazugehörigen Antragsverfahren mit Stand August 2022 geschildert werden.

## Auswirkungen der CTR auf Prozesse im Lebenszyklus einer klinischen Prüfung

Da Änderungen in der Prozesslandkarte notwendig werden, empfiehlt es sich, sogenannte Gap-Analysen durchzuführen, um die erforderlichen Anpassungen zu ermitteln und die daraus resultierende Ressourcenplanung vorzunehmen. Dies kann z. B. im Rahmen einer einfachen Übersichtstabelle erfolgen, die die Anforderungen der CTR den jeweils betroffenen Systemen bzw. Strukturen (z. B. Standardarbeitsanweisungen – SOPs, Verträge, Pharmakovigilanz) sowie den involvierten verantwortlichen Parteien (z. B. Qualitäts‑, Projektmanagement oder Biometrie) zuordnet. Nach der Ermittlung des Anforderungsprofils können ein entsprechender Maßnahmenkatalog erarbeitet, eine Priorisierung vorgenommen und Verantwortlichkeiten für die Umsetzung zugewiesen werden. Der gesamte Prozess wird vom Qualitätsmanagement des (akademischen) Sponsors begleitet und unterstützt. Die gleichzeitige Durchführung von klinischen Prüfungen nach „alter“ EU-Richtlinie [4] und gemäß der aktuellen CTR erfordert von den Beteiligten ein sicheres Wissen und somit SOPs in beiden Rechtsrahmen. An der Studienzentrale des Universitätsklinikums Bonn waren z. B. ca. 20 % der vorhandenen SOPs von einer Anpassung betroffen. Darüber hinaus erfordern völlig neue Prozesse, wie die Nutzerrechte-Verwaltung in CTIS, die Erstellung zusätzlicher SOPs.

Aus den neuen und geänderten rechtlichen Anforderungen resultiert auch die Notwendigkeit, bestehende Verträge und Vertragsvorlagen entsprechend zu adaptieren. Beispiele dafür sind Verträge zur Übernahme der Sponsorenschaft mit dem (Haupt‑)Prüfer/Sponsorvertreter, Verträge mit den Prüfstellen oder Verträge mit sonstigen eingebundenen Einrichtungen (sogenannte Vendoren wie Apotheke, Labor, Auftragsforschungsinstitute etc.). Auch hier entstehen große Herausforderungen für die akademischen Rechtsabteilungen, die häufig mit knappen Personalressourcen arbeiten müssen.

Fast alle Anforderungen, die bislang nur für Prüfpräparate/Placebo galten, betreffen nun auch Hilfspräparate (Auxiliary Medicinal Products; beispielsweise Arzneimittel, die als Hintergrundtherapie oder Provokationssubstanz eingesetzt bzw. zur Bewertung der Endpunkte in der klinischen Prüfung verwendet werden). Dies ist eine wesentliche Neuerung der CTR. So müssen nunmehr auch für Hilfspräparate im Antragsdossier Angaben zur pharmazeutischen Qualität und Sicherheit gemacht werden, Etikettierungsvorgaben sind zu berücksichtigen, Anforderungen an die Zulassung und Einfuhr sind mit denen für Prüfpräparate gleichgesetzt. Auch in dieser Hinsicht sind diverse SOPs/Verfahrensvorschriften betroffen, die aktualisiert werden müssen.

Ein weiterer neuer Aspekt ist das Erfordernis, nicht in der EU zugelassene Prüf- und Hilfspräparate in der Arzneimitteldatenbank „eXtended EudraVigilance Medicinal Product Dictionary“ (XEVMPD; [5]) zu registrieren. Sofern also in einer klinischen Prüfung ein Prüfpräparat, Hilfspräparat oder (kommerziell erhältliches) Placebo zum Einsatz kommen soll, das keine Zulassung in der EU hat, muss durch den Sponsor eine Registrierung in der oben genannten Datenbank vorgenommen werden, damit das Prüfprodukt im EU-Antragsdossier angezeigt und im Rahmen der Antragstellung hochgeladen werden kann. Für die Zuteilung eines Nutzerzugangs in der XEVMPD-Datenbank ist die Absolvierung eines Trainings einschließlich Abschlusstests erforderlich. Sponsoren bzw. Koordinierungszentren ohne eigene Pharmakovigilanzabteilung müssen diese Ressource künftig aufbauen bzw. delegieren.

Neu eingeführt wird das Konzept der minimalinterventionellen klinischen Prüfung („low interventional clinical trial“), das gerade im Rahmen von wissenschaftsinitiierten klinischen Prüfungen („investigator-initiated trials“ – IIT) zutreffen kann. Mit der Einstufung einer klinischen Studie als minimalinterventionelle klinische Prüfung soll dank des risikobasierten Ansatzes eine Reduzierung des Aufwands bei der Planung und Durchführung ermöglicht werden (z. B. in Hinblick auf Dokumentation im Rahmen der Good Manufacturing Practice (GMP); [6]). Noch ist nicht ausreichend klar, worin diese Vereinfachungen bestehen werden. Beispielsweise ist gemäß Art. 76 CTR keine separate Probandenversicherung für minimalinterventionelle klinische Prüfungen erforderlich, sofern bereits ein anwendbares Entschädigungssystem vorhanden ist. Als „anwendbares Entschädigungssystem“ ist beispielsweise eine Betriebshaftpflichtversicherung anzusehen, deren Versicherungsschutz die Durchführung, Planung und Organisation von klinischen Studien umfasst.

Relevante Studiendokumente, wie Prüfplan und Patienteninformation/Einwilligungserklärung oder Qualifikationsunterlagen der Prüfer werden nun veröffentlicht und müssen von den Sponsoren für diesen Zweck zusätzlich in anonymisierter Form im CTIS hochgeladen werden. Auch hinsichtlich der Berichterstattung klinischer Prüfungen gibt es eine relevante Änderung: Zur Erhöhung der Transparenz von Forschungsergebnissen gegenüber den Teilnehmenden einer klinischen Prüfung sowie der interessierten Öffentlichkeit sind die Ergebnisse jeder klinischen Prüfung in laienverständlicher Sprache zusammenzufassen („lay summary“). Die Anforderungen sind in der entsprechenden Guidance „Good Lay Summary Practice“ [7] formuliert.

Durch die CTR wurde eine Archivierungsdauer von 25 Jahren neu festgelegt. Bisher galten bei IIT die üblichen 10 Jahre (Ausnahmen vorbehalten). Es bedarf noch der Klärung, wie dies im Einklang damit steht, dass die Patientenakten der Prüfungsteilnehmenden gemäß nationalem Recht aufbewahrt werden, da diese Fristen, zumindest in Deutschland, erheblich kürzer sind. Akademische Sponsoren, die u. a. aus Kostengründen noch überwiegend mit papierbasierter Studiendokumentation arbeiten, müssen somit langfristige Archivierungskapazitäten aufbauen bzw. finanzieren, was sich über zeitlich eng limitierte öffentliche Fördermittel kaum abbilden lässt.

## Aspekte der Sponsorverantwortung

Die CTR führt neu das optionale Konzept der Co-Sponsorenschaft ein, d. h., mehrere europäische Einrichtungen können gemeinsam mit vertraglich klar abgegrenzten Verantwortlichkeiten eine klinische Prüfung durchführen. Weiterhin sind auch hier die (Co‑)Sponsoren in ihrer umfassenden Überwachungsfunktion gefragt. Insbesondere für die akademischen Forschungseinrichtungen ergeben sich hieraus Chancen für internationale Kooperationen und solche mit der (Pharma‑)Industrie, z. B. durch abgegrenzte Verantwortlichkeiten in beteiligten europäischen Mitgliedstaaten.

Ebenfalls entsteht durch die CTR neu die Verpflichtung der Sponsoren zur Meldung von „schwerwiegenden Verstößen“ („serious breaches“) über CTIS, die die Sicherheit der Studienteilnehmenden oder die Datenqualität erheblich gefährden (können). Das sind beispielsweise Abweichungen von der CTR, der „Guideline for good clinical practice“ (ICH GCP E6; [8]) oder dem Prüfplan, aber auch Liefer- oder Qualitätsprobleme bei Prüfpräparaten. Bisher wurden solche Abweichungen zwar ebenfalls dem Sponsor gemeldet und strukturiert bearbeitet, eine Meldung an die EMA [9] innerhalb definierter Fristen gab es aber mit Ausnahme der Länder Vereinigtes Königreich und Spanien nicht. Die bei den Sponsoren im Qualitätsmanagement bereits existierenden Prozesse und Berichtsstrukturen, die nun die neuen, engen Fristen berücksichtigen sollen, müssen entsprechend aufgebaut bzw. angepasst werden. Insbesondere ist die Schulung aller involvierten Parteien, Vendoren und Prüfstellen bei Studieninitiierung und anlassbezogen durch den Sponsor hier von hoher Relevanz.

## Auswirkungen auf die Pharmakovigilanz

Meldungen von Verdachtsfällen unerwarteter schwerwiegender Nebenwirkungen (*Suspected Unexpected Serious Adverse Reaction* – SUSAR) sollen in Zukunft nur noch über das Informationsnetzwerk und Managementsystem „EudraVigilance“ der EMA erfolgen. Vorgaben zu separaten SUSAR-Meldungen an Ethikkommissionen und Prüfer entfallen, was zu einem einheitlichen und verschlankten Meldewesen und somit verringertem Aufwand führt. Die Praxis wird zeigen, wie die Informationsweiterleitung an die Prüfer zu gestalten ist, beispielsweise durch Sammelmeldungen mit Bewertung. Aus Sicht der Pharmakovigilanz sind die Anforderungen zu unerwarteten Ereignissen mit Auswirkungen auf das Nutzen-Risiko-Verhältnis (Art. 53 CTR), Schwangerschaften und sogenannte „special situations“ (z. B. Medikationsfehler) nicht klar in der CTR definiert.

## Clinical Trials Information System (CTIS)

Ein wichtiger Schritt zur Implementierung der Anforderung der CTR ist für die akademischen Sponsoren die Entscheidung über die zukünftige Nutzerstruktur im CTIS. Das Portal sieht für Sponsoren 2 Möglichkeiten der Nutzerverwaltung vor. Der organisationszentrierte Ansatz („organisation-centric approach“) ist für Sponsoren gedacht, die regelmäßig klinische Prüfungen im CTIS verwalten. Hierzu ist es nötig, dass sich ein sogenannter High-Level-Administrator bei der EMA registriert, der weitere Rollen (z. B. CT Admin, Part II Preparer) zuweisen kann. Vorab ist die Registrierung im Organisation Management System (OMS; [10]) der EMA Voraussetzung. Bei diesem Ansatz bleibt die Hoheit über die Nutzerverwaltung bei der Organisation. Der Sponsor hat so jederzeit den Überblick, welche klinischen Prüfungen an seinem Standort in CTIS angelegt sind. Für den Aufbau von Rollen und Prozessen (Beantragung von Rollen, Organisations-Identifikator (ORG-ID, Prüfstellen LOC ID) etc.) sowie die Verzahnung mit existierenden Sponsorprozessen werden zusätzliche personelle Ressourcen benötigt.

Für akademische Sponsoren oder kleine bis mittelständische Unternehmen, die wenige klinische Prüfungen in eigener Verantwortung durchführen, kann der studienzentrierte Ansatz („trial-centric approach“) geeignet sein. Dieser Ansatz ermöglicht es jedem Nutzer mit einem EMA-Zugang, eine klinische Prüfung im Auftrag des Sponsors im System anzulegen. Die Nutzerverwaltung findet hier ausschließlich auf Studienebene statt, sodass dieser Ansatz deutlich weniger Übersicht für den Sponsor bietet. Ein Wechsel der Nutzerverwaltungsstruktur ist aber jederzeit möglich.

Grundsätzlich sind Unterstützungsangebote der EMA zu CTIS und OMS sehr umfangreich [11]. Ein Helpdesk sowie eine Vielzahl an Schulungs- und Informationsmaterialien ermöglichen den Sponsoren die Einarbeitung in das neue System und die aktuellen Anforderungen. Ein mehrtägiger Schulungsaufwand ist hierbei einzuplanen.

**Figure 1:**
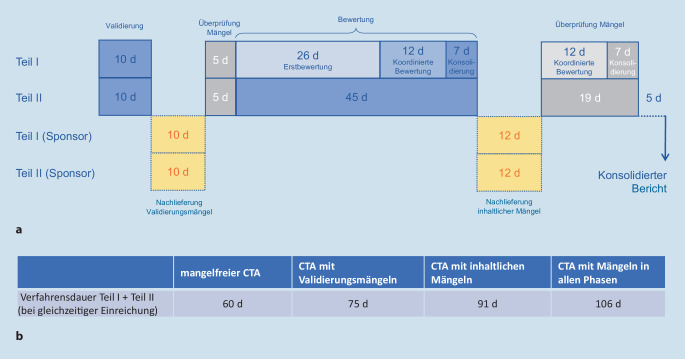

Positiv zu erwähnen ist die Funktion „Timetable“ im CTIS. Diese Übersicht stellt die einzelnen Phasen übersichtlich dar und ermöglicht eine relativ gute Planung der internen Abläufe, insbesondere im Hinblick auf Personalressourcen für die Bearbeitung der Rückfragen („request for information“ – RFI). Insgesamt ermöglicht CTIS dem Sponsor durch den „Timetable“, aber auch durch weitere Übersichten wie den „MSC trial status“ („member state concerned“ – MSC; Studienstatus beteiligte Mitgliedstaaten) eine gute Nachverfolgbarkeit der einzelnen Schritte des Genehmigungsprozesses. Eine Übersicht der Fristen und des Prozessflusses ist in Abb. 1 dargestellt.

Am Standort Dresden wurde eine EU-geförderte klinische Prüfung in 6 Mitgliedstaaten der EU in CTIS eingereicht, am Standort Bonn eine deutsche monozentrische Studie. Die Dauer bis zur Genehmigung betrug im Fall der multinationalen IIT 117 Tage. Im Fall der monozentrischen klinischen Prüfung wurden die Antragstellenden durch die Ethikkommission nach 88 Tagen aufgefordert, den Antrag zunächst zurückzuziehen und mit einem geänderten Einschlusskriterium erneut einzureichen. Ab dem Zeitpunkt der Neueinreichung bis zur Genehmigung der klinischen Prüfung vergingen 49 Tage. Die bei den Einreichungen gesammelten Erfahrungen sind in Tab. 1 dargestellt.

**Table 1:** 

Problem	Problembeschreibung	Mögliche Maßnahmen
Keine direkte Kommunikation mit Behörden und Ethikkommissionen (EK)	Die direkte Kommunikation mit Behörden und EK ist in CTIS nicht möglich, sodass Probleme nicht unkompliziert und zeitnah gelöst werden können	Die deutschen Bundesoberbehörden (BOB) und die EK bieten nun eine kostenpflichtige Vorabkonsultation an, im Rahmen derer die für den Genehmigungsprozess zuständige EK (gemäß Geschäftsverteilungsplan [12]) bekannt wird. Somit ist ein direkter Kontakt zu BOB und EK hergestellt, der auch im Verlauf der Einreichung genutzt werden kann
	*Beispiel:* Bei einer Rückfrage zu einem Einschlusskriterium wichen die Meinungen zur Auslegung einer Behandlungsleitlinie durch Antragsteller und EK voneinander ab. Bedingt durch das Verfahren wurde dem Antragsteller geraten den Antrag zurückzuziehen und in überarbeiteter Fassung erneut einzureichen, um eine Versagung des Antrags zu vermeiden. Eine „Genehmigung mit Auflagen“ hätte eine Alternative zur Neueinreichung dargestellt	Volle Nutzung der Option „Genehmigung mit Auflagen“ durch EK und Behörden
Englisch als Fremdsprache	Für (fast) alle Beteiligten waren die englischsprachigen Rückfragen (RFI) der Behörden oder EK teilweise nicht klar verständlich. Es besteht die Gefahr, dass falsche Antworten gegeben werden	Die Rückfragen sollten zusätzlich in die jeweilige Landessprache des Bearbeitenden (in der Behörde bzw. EK) übersetzt werden
Unvollständige Harmonisierung	Die niederländische wie auch die spanische EK verlangten die Verwendung eigener, teilweise den EudraLex Volume 10 ähnlichen Vorlagen	Es sollte ein EU-weiter Abgleich von eingeforderten Unterlagen, verwendeten Begrifflichkeiten sowie Standards stattfinden und somit eine weitere Harmonisierung. Der Austausch der für die Bearbeitung von Teil-II-verantwortlichen Stellen sollte verstärkt werden
	Begriffe werden unterschiedlich verwendet: „principal investigator“, „investigator“, „medical member of the investigating team“ (ungleich „investigator“)	
	In Deutschland verlangt die EK weiterhin Qualifikationsnachweise aller Prüfer (nicht nur des Hauptprüfers), sofern diese im „Site Suitability Form“ aufgeführt sind	Harmonisierung von Begrifflichkeiten und geforderten Unterlagen unter den Ländern der EU
	In Deutschland muss jeder Prüfer mit vollständiger Autorisierung für alle Aufgaben als „licensed physician with specialist standard in the indication“ bezeichnet werden. Andere Beschreibungen werden nicht akzeptiert	
	In den Ländern wird mit möglichen Gebührenreduktionen unterschiedlich umgegangen	Die Erstellung einer Gebührenübersicht für alle Mitgliedstaaten und mögliche Gebührenermäßigungen (inkl. Auflistung der hierfür einzureichenden Unterlagen) wäre hilfreich
Umgang mit Rückfragen (RFI)	Die bereits engen Bearbeitungsfristen können willkürlich gekürzt werden	Die Mindestbearbeitungsfristen sollten nicht weiter gekürzt werden dürfen
	Zur Beantwortung von Rückfragen stehen dem Antragsteller theoretisch 12 Tage zur Verfügung, was auch Dokumente betreffen kann, die in der Clinical Trials Regulation (CTR) als „optional“ gekennzeichnet sind	Eine technische Lösung für die verbesserte Bearbeitung von parallel gestellten Rückfragen wäre hilfreich
	Das gleichzeitige Stellen von 2 Rückfragen erschwerte die Beantwortung, weil jeweils eine neue Draftversion des Antragsdossiers erstellt werden musste	Rückfragen sollten inhaltlich abgestimmt werden
	Teilweise kam es zur inhaltlichen Dopplung von Rückfragen aufgrund der fehlenden Abstimmung zwischen den Mitgliedstaaten	
Technische Probleme mit CTIS	Nach der geforderten Anpassung von länderspezifischen Dokumenten im Rahmen von Teil 1 des regulatorischen Prozesses konnten die länderspezifischen Sektionen nicht noch einmal geöffnet werden, um die aktualisierten Dokumente an der richtigen Stelle hochzuladen	Eine technische Anpassung von CTIS ist notwendig
	CTIS hat selbstständig eine Frist für eine Rückfrage definiert, die in der Vergangenheit lag, was zeitweise den gesamten Antragsprozess des eingereichten Antrags gefährdete	Es sollte eine umfängliche und auch auf besondere Fälle angepasste CTIS-Eingabekonvention für Antragstellende erstellt werden
	An den Antragsteller geht keine automatische Information, wenn beispielsweise Rückfragen im CTIS gestellt werden, die fristgerecht beantwortet werden müssen	
	Antragstellenden ist nicht klar, an welcher Stelle bestimmte Dokumente hochgeladen werden sollen	
	*Beispiel:* Im Fall, dass ein Medizinprodukt oder ein In-vitro-Diagnostikum (IVD; Bluttest o. Ä.) in der klinischen Prüfung zur Anwendung kommt und dies nicht im Sinne einer Konformitätsbewertung oder klinischen Leistungsbewertung geschieht, ist unklar, an welcher Stelle die entsprechenden Unterlagen hochgeladen werden müssen	

## Diskussion

Die Bündelung sämtlicher regulatorischer Verpflichtungen und die einheitliche Digitalisierung im CTIS sind insgesamt als positiv hervorzuheben. Es entfällt die separate Beantragung einer Studiennummer, da diese „EU CT number“ mit dem Anlegen der klinischen Prüfungen im CTIS automatisch generiert wird. Weiterhin entfallen die ehemaligen Meldungen nach § 67 Arzneimittelgesetz (AMG; [13]) zu Sponsor und Prüfstellen bei Studienbeginn, Änderungsanzeigen, Unterbrechung und Ende der klinischen Prüfung. Die Einreichung der jährlichen Sicherheitsberichte („annual safety reports“ – ASR, ehemals „development safety update reports“ – DSUR) erfolgt ebenfalls über CTIS.

In Deutschland war die Vorgabe der EU-Richtlinie zur Veröffentlichung der Studienergebnisse im EU-Clinical Trials Register (EUCTR; [14]) nicht eindeutig aus dem Arzneimittelgesetz ersichtlich [15], sodass in den vergangenen Jahren, insbesondere im akademischen Bereich, ein zunächst niedriger Anteil von klinischen Prüfungen in EUCTR veröffentlich worden war. Akademische Sponsoren hatten große Anstrengungen unternommen, um diesem Problem Abhilfe zu leisten. Nun ist aber durch die CTR zum einen Rechtssicherheit in Bezug auf die Veröffentlichungspflicht geschaffen, zum anderen erfolgt das Berichtswesen direkt in CTIS, wodurch die Anzahl anzuwendender Systeme und der Arbeitsaufwand für die (akademischen) Nutzer verringert wird. Im „organization-centric approach“ ist eine Übersicht über alle beantragten und laufenden klinischen Prüfungen für die Sponsoren möglich.

Die Leistung der EMA in Bezug auf die Erstellung von didaktisch sehr gut aufbereitetem und umfangreichem Schulungsmaterial hinsichtlich CTIS und OMS ist beachtlich, genau wie die regelmäßige Aktualisierung von FAQs und die allgemeine Informationsweitergabe. Hierbei ist allerdings anzumerken, dass die bereits knappen Ressourcen akademischer Koordinierungszentren oder Studienzentralen, die benötigt werden, um die Aktualisierungen und FAQs zu verinnerlichen, künftig noch stärker beansprucht werden. Wünschenswert wäre ein umfassendes Wissensmanagementsystem zu CTIS und zur CTR.

Kosten einzelner klinischer Prüfungen sind in Bezug auf die generelle Durchführbarkeit von IITs in akademischen Forschungseinrichtungen oft ein entscheidendes Kriterium. Durch die Überführung laufender Projekte in die CTR entstehen ungeplante Mehrkosten. Bei neu beantragten Projekten treiben zum einen die verlängerte Archivierungsdauer von 25 Jahren, zum anderen die schlecht planbaren regulatorischen Gebühren die Gesamtkosten von IITs in die Höhe. Zusammen mit den stets steigenden Anforderungen im Rahmen klinischer Prüfungen führt dies zu einer Kostensteigerung, die einen Einfluss auf die Förderquoten bei öffentlichen Mittelgebern hat und damit möglicherweise zu einer geringeren Anzahl durchgeführter IITs in Deutschland führt.

Im CTIS sind sämtliche Unterlagen – sowohl die eingereichten als auch die, die die Genehmigung dokumentieren – enthalten. Diese wurden bisher im Studienordner (Trial Master File) beim Sponsor, aber auch in den Prüfzentren u. a. zur Einsichtnahme von Inspektionsteams abgelegt. Im Hinblick auf einen sinnvollen Umgang mit personellen und auch Rohstoffressourcen sollte nun überlegt werden, ob dies noch adäquat und zwingend notwendig ist, da die Behörden und damit die Inspektionsteams Zugang zu den elektronischen Dokumenten in CTIS haben.

## Fazit

Akademische Sponsoren können und werden die notwendigen Anpassungen, die sich aus der CTR ergeben, vornehmen. Die ersten erfolgreichen Einreichungen je einer nationalen und einer internationalen IIT in CTIS sind bereits erfolgt. Zwar ist eine EU-weite Harmonisierung der Anforderungen noch nicht vollzogen, viele technische Mängel müssen noch behoben werden, mittel- und langfristig werden aber Erleichterungen in Bezug auf regulatorische Prozesse auch für akademische Sponsoren spürbar sein. Hier sind intensivierte Absprachen mit nationalen Oberbehörden und Ethikkommissionen, effektives Wissensmanagement und eine verbesserte Kommunikation, ggf. unter Nutzung entsprechender Systeme gefragt. Bei der Beantragung von Fördermitteln für IITs muss der zusätzliche Aufwand, der durch die gestiegenen Anforderungen in verschiedenen Bereichen (Verträge, „schwerwiegende Verstöße“, Prüf- und Hilfspräparate, laienverständliche Zusammenfassung, Archivierung etc.) bedingt ist, berücksichtigt werden, was die Kosten für IITs weiter in die Höhe treiben wird und somit die Zahl der IITs in Deutschland reduzieren könnte.

## Footnotes

1 Als Ende einer klinischen Prüfung gilt üblicherweise die letzte Visite des letzten Studienteilnehmenden, es sei denn, der Prüfplan definiert das Ende der klinischen Studie anders.

2 GCP-V: „Verordnung über die Anwendung der Guten klinischen Praxis bei der Durchführung von klinischen Prüfungen mit Arzneimitteln zur Anwendung am Menschen“.
